# Akt and SHIP Modulate *Francisella* Escape from the Phagosome and Induction of the Fas-Mediated Death Pathway

**DOI:** 10.1371/journal.pone.0007919

**Published:** 2009-11-20

**Authors:** Murugesan V. S. Rajaram, Jonathan P. Butchar, Kishore V. L. Parsa, Thomas J. Cremer, Amal Amer, Larry S. Schlesinger, Susheela Tridandapani

**Affiliations:** 1 Department of Internal Medicine, The Ohio State University, Columbus, Ohio, United States of America; 2 The Ohio State Biochemistry Program, The Ohio State University, Columbus, Ohio, United States of America; 3 Molecular, Cellular, and Developmental Biology Program, The Ohio State University, Columbus, Ohio, United States of America; 4 Center for Microbial Interface Biology, The Ohio State University, Columbus, Ohio, United States of America; University of Cambridge, United Kingdom

## Abstract

*Francisella tularensis* infects macrophages and escapes phago-lysosomal fusion to replicate within the host cytosol, resulting in host cell apoptosis. Here we show that the Fas-mediated death pathway is activated in infected cells and correlates with escape of the bacterium from the phagosome and the bacterial burden. Our studies also demonstrate that constitutive activation of Akt, or deletion of SHIP, promotes phago-lysosomal fusion and limits bacterial burden in the host cytosol, and the subsequent induction of Fas expression and cell death. Finally, we show that phagosomal escape/intracellular bacterial burden regulate activation of the transcription factors sp1/sp3, leading to Fas expression and cell death. These data identify for the first time host cell signaling pathways that regulate the phagosomal escape of *Francisella*, leading to the induction of Fas and subsequent host cell death.

## Introduction

*Francisella tularensis* is a highly virulent, gram negative, intracellular bacterium responsible for the zoonotic disease tularemia. There are four closely related subspecies of *F. tularensis*: *tularensis* (type A), *holarctica* (type B), *mediasiatica* and *novicida*. The type A subspecies *F. tularensis tularensis* and type B subspecies *F. tularensis mediasiatica* are classified as a Category A agents because they are potential agents of biowarfare with a low dose required for lethality [1], [2]. *F. tularensis* subsp. *novicida* U112 is an environmental isolate which is highly virulent in mice but attenuated in humans [3]. Most importantly, *F. tularensis* subsp. *novicida* has a similar intracellular lifestyle in monocytes/macrophages to the virulent *F. tularensis* subsp. *tularensis* [4], [5], thus serving as a good model to understand the molecular mechanisms of *Francisella*-induced host responses.

*Francisella* infects and replicates within phagocytic cells of the immune system such as monocytes and macrophages [1]. Host cell entry occurs by looping phagocytosis [6]. Shortly after phagosome biogenesis there is maturation to an early endosome and limited acquisition of late endosomal markers. After acidification of the vacuole there is escape from the phagosome [5]. While the precise timing of escape has been debated, it has been reported that during the first hour after host cell entry there is evasion of phago-lysosomal fusion and escape into the host cell cytosol [7], [8]. This phagosomal escape is dependent on the *Francisella* pathogenicity island proteins (*iglC*, *iglD* etc.) and its regulator *mglA* [9], [10]. Post phagosomal escape, *Francisella* replicates in the cytosol and induces apoptosis of the host cell. A series of studies by Lai et al. demonstrated that the induction of apoptosis in the J774.1 macrophage cell line infected with *F. tularensis* LVS required bacterial escape and replication. It was further demonstrated that under these conditions there is release of cytochrome-C and the activation of caspase-3 [11]–[13]. In a recent study, Mariathasan et al demonstrated a role for caspase-1 in *Francisella*-induced apoptosis [14]. However, the upstream pathways leading to release of cytochrome-C and activation of the caspases are not yet clearly understood.

The host cell response to infection includes the production of a number of pro-inflammatory cytokines such as TNFα, IL-1β, IL-12 and IL-23 [14]–[19]. These cytokines in turn promote the production of IFNγ from NK cells and T cells. Several studies have shown that IFNγ is protective during *Francisella* infection [4], [20], [21]. Although, phagosomal escape has been shown to be required for the activation of caspase-1 and the release of IL-1β [14,16], it is also associated with a suppression of pro-inflammatory cytokine production [15], [22]. We have recently demonstrated that pro-inflammatory cytokine production in infected cells is promoted by the PI3K/Akt pathway and is negatively regulated by the inositol phosphatase SHIP [17], [22].

In the present study we analyzed the molecular mechanism of *Francisella-*induced apoptosis and report that Fas expression is enhanced in infected macrophages leading to cell death. Further, we show that Fas recruits the DISC components (FADD and caspase-8). Fas expression is dependent on phagosomal escape of the pathogen. Using pharmacological inhibition, overexpression, and transgenic mouse models, we demonstrate that Akt negatively regulates Fas expression. In addition, although Akt did not influence uptake of the bacteria, it promoted significant bacterial clearance and enhanced phago-lysosomal fusion. Consistently, *Francisella-*infected SHIP-deficient macrophages displayed lower Fas expression and significantly enhanced phago-lysosomal fusion events. Therefore, we propose that host cell phosphoinositide signaling influences the intracellular lifestyle of *Francisella* modulating phagosomal escape/intracellular bacterial burden and the subsequent induction of Fas expression and host cell death.

## Materials and Methods

### Cells, Antibodies and Reagents

RAW 264.7 cells were obtained from American Type Culture Collection (Manassas, VA) and cultured in RPMI-1640 (Gibco-BRL, Rockville, MD) supplemented with 5% heat-inactivated fetal bovine serum (FBS), L-glutamine and antibiotic cocktail of penicillin (10,000 U/mL) and streptomycin (10,000 ug/mL). Antibodies specific for phospho-Serine Akt, Caspase-8 and Caspase-3 were purchased from Cell Signaling Technology (Boston, MA). Actin and Akt antibodies were purchased from Santa Cruz Biotechnology (Santa Cruz, CA). Fas (Clone 7C10) antibody was purchased from Upstate Cell Signaling Solutions (Charlottesville, VA). Rabbit polyclonal SHIP antibody was a generous gift from Dr. K. Mark Coggeshall (Oklahoma Medical Research Foundation, Oklahoma City, Oklahoma, United States). The PI3K inhibitor LY 294002 was purchased from Calbiochem (La Jolla, CA).

### Bacterial Strains and Culture

*F. tularensis* subsp. *novicida* strain U112 (JSG1819), and an *mglA* mutant of *F. tularensis* subsp. *novicida* U112 (JSG2250) were provided by Dr. John S. Gunn (The Ohio State University, Columbus, OH). All *Francisella* strains were cultured on Chocolate II agar plates (BD Biosciences, San Jose, CA) at 37°C.

### Culture of Murine Bone Marrow Macrophages (BMM)

Transgenic mice with macrophage-specific expression of a constitutively-active form of Akt, Myr Akt, were provided by Dr. Michael C. Ostrowski (The Ohio State University, Columbus, OH) and have been previously described [23]. SHIP-deficient animals were generously provided by Dr. G. Krystal (BC Cancer Agency, Vancouver, British Columbia, Canada). BMM were isolated and cultured as previously described [24]. Briefly, bone marrow cells were collected by flushing the femurs of mice with RPMI and cultured in 10 cm tissue culture dishes in RPMI containing 10% FBS plus 10 µg/ml polymixin B and supplemented with 20 ng/ml CSF-1 for 7 days. BMM derived in this manner were >99% positive for Mac-1, as determined by Flow Cytometry.

### Microarray Analysis

BMM were isolated and differentiated from three C57/Bl6 mice as described previously [24]. Seven days after differentiation, macrophages from each mouse were plated into 2 wells of a 6-well dish at 2.5 million per well in RPMI containing 5% heat-inactivated FBS. *F. tularensis* subsp. *novicida* was added to each well at an MOI of 100. Cells were gently mixed and incubated at 37°C in 5% CO_2_ for 24 h. RNA was extracted using TRIzol Reagent (Invitrogen Life Technologies, Carlsbad, CA), column-purified using RNeasy columns (Qiagen, Valencia, CA) and then hybridized to GeneChip® Mouse Genome 430 2.0 Arrays (Affymetrix, Santa Clara, CA). Expression values were calculated using the “gcrma” package in BioConductor (www.bioconductor.org) and the “limma” package [25] was used to find genes significantly different (p≤0.05) between infected and uninfected samples.

### Infection, Lysis, and Western Blotting

BMM and RAW 264.7 cells were infected with either *F. tularensis* subsp. *novicida* or the *mglA* mutant at an MOI of 100, except in the dose response experiment. The plates were kept on a rocker for five minutes, centrifuged at ∼650×g for 4 minutes at room temperature and then incubated at 37°C in the presence of 5% CO_2_ for the indicated time points. The infected and uninfected cells were lysed in TN1 buffer (50 mM Tris (pH 8.0), 10 mM EDTA, 10 mM Na_4_P_2_O_7_, 10 mM NaF, 1% Triton X-100, 125 mM NaCl, 10 mM Na_3_VO_4_, 10 µg/ml each aprotinin and leupeptin), incubated on ice for 10 minutes and then centrifuged at 13,000 rpm at 4°C to clear the nuclei. Protein concentrations were measured using the Bio-Rad DC protein assay kit (Bio-Rad Laboratories, Hercules, CA, USA). Cell lysates were boiled in Laemmli sample buffer and were separated by SDS-PAGE, transferred to nitrocellulose filters, probed with the antibody of interest, and developed using ECL (Amersham Biosciences, Pittsburg, PA, USA).

### Immunoprecipitation of DISC

RAW 264.7 cells were infected with *F. tularensis* subsp. *novicida* (100 MOI) and incubated for 24 h at 37°C. Cells were lysed in TN1 buffer (as described above), and post-nuclear lysates were subjected to immunoprecipitation with Fas antibody. The immunoprecipitated complexes were separated by SDS-PAGE, transferred to nitrocellulose filters, probed with FADD, caspase-8 or Fas antibodies and developed using ECL.

### Western Blot Data Quantification

The ECL signal was quantified using a scanner and a densitometry program (Scion Image), as previously described [23], [26]. To quantify the specific signal in the infected samples, we first subtracted the background, normalized the signal to the amount of actin in the lysate, and plotted the values as percent increase over uninfected samples, as previously described. All data were analyzed by a student's t-test and a p value of less than 0.05 was considered significant.

### Transfection

RAW 264.7 cells were transfected with the appropriate plasmid DNA using the Amaxa Nucleofector (Amaxa Biosystems, Gaithersburg, MD,USA) as previously described [23]. Briefly, 5×10^6^ cells were resuspended in 100 µl Nucleofector Solution, then 5 ug of empty vector (pCMV6) or plasmid containing wild-type Akt (a kind gift from Dr. P. Tsichlis, Fox Chase Cancer Center, Philadelphia, PA, USA) was nucleofected according to the manufacturer's instructions. Cells were then seeded in 6-well plates containing 1.5 ml of RPMI supplemented with 5% FBS and incubated for 12 h in a CO_2_ incubator at 37°C. Transfected cells were either left uninfected or were infected with *F. tularensis* subsp. *novicida* for different time periods (3 h, 6 h, 12 h and 24 h). Cells were lysed in TN1 lysis buffer and lysates were analyzed.

### Colony Forming Unit Assays

Wild type and Myr Akt BMM were infected with *F. tularensis* subsp. *novicida* (100 MOI) for the different time points (30 min, 2 h, 5 h and 9 h). After infection, cells were washed two times and incubated with 50 µg/ml of gentamicin for 30 minutes at 37°C in 5% CO_2_. The cells were subsequently washed twice and lysed in 0.1% SDS for 5 minutes. Immediately, 10-fold serial dilutions were made and appropriate dilutions were plated on Chocolate II agar plates. Assays were performed in triplicate for each test group.

### Measurement of Bacterial Burden in Organs

Enumeration of organ burden was performed as previously described with a few modifications [27]. Briefly, mice were infected with 200 CFU of *F. tularensis* subsp. *novicida* by intraperitoneal injection. After 48 h mice were sacrificed and organs (spleen, lung and liver) were removed under aseptic conditions. The isolated organs were washed twice in sterile PBS and homogenized in 2 ml of sterile PBS using a tissue homogenizer (Tissue-Tearor, BioSpec Products Inc, Bartlesville, OK, USA). Immediately, the suspension was serially diluted (10 fold at each step) in PBS. Appropriate dilutions were plated on chocolate agar plates in triplicate and incubated at 37°C overnight. The number of bacterial colonies on each plate was counted and bacterial numbers were expressed as log_10_ CFU per mg of the organ. All mouse experiments were performed with ILACUC-approved protocols.

### Cytopathogenicity Assay (LDH Assay)

*F. tularensis* subsp. *novicida* induced cell death was monitored using the Lactate Dehydrogenase-based In-Vitro Toxicology Assay kit (Sigma, Saint Louis, Missouri, USA). Briefly, 1 million RAW 264.7 cells or BMMs were seeded in 12-well plates and allowed to adhere. Next, the cells were infected with *F. tularensis* subsp. *novicida* (100 MOI) for the indicated time points. Cell supernatants were collected, and cells were lysed with the LDH assay lysis solution provided with the kit. Percent LDH release was calculated using the formula: [(absorbance of supernatant/absorbance of supernatant + absorbance of lysate)*100]. All experiments were performed at least three times, each time in triplicate.

### FCP-LAMP-1 Acquisition Analysis

BMM (0.3×10^−6^) were plated on glass coverslips in 12-well culture plates overnight. Synchronized infections of *F. tularensis* subsp. *novicida* were performed at an MOI of 100. After 30 min of infection cells were washed twice in PBS and treated with gentamicin for 30 min and incubated for an additional 2 h. Cells were then fixed with freshly prepared periodate-lysine-paraformaldehyde fixative containing 5% sucrose [28], [29] for 20 minutes at 37°C. The cells were then rinsed with PBS and permeabilized with ice cold methanol. Antibodies were diluted in PBS with 5% goat serum (PBSGS). The anti-LAMP-1 (1D4B; Developmental Hybridoma Bank) was diluted 1∶100 in PBSGS and the secondary antibody Oregon Green® 488 goat anti-rat IgG (Invitrogen Life Technologies, Carlsbad, CA, USA) was diluted 1∶250 in PBSGS. All antibody incubations were performed for a period of one hour at 37°C. Macrophage and bacterial nuclei were labeled with 0.1 µg of the DNA stain 4,6-diamidino-2-phenylindole (DAPI; Molecular Probes, Carlsbad, CA, USA) per ml in PBS for 5 minutes at room temperature. The coverslips were mounted using ProLong Gold Antifade Reagent (Invitrogen Life Technologies, Carlsbad, CA, USA). The co-localization of *F. tularensis* subsp. *novicida* with LAMP-1 was analyzed by confocal microscopy. Images were acquired using a Zeiss Laser Scanning Microscope LSM 510. FCP-lysosome fusion events were considered positive only when a complete ring of intensified LAMP1 staining was detected around the bacterium. Three independent, blinded evaluations were performed for each sample.

### LysoTracker Co-Localization with *F. tularensis* Subsp. *novicida*

BMM were plated on glass coverslips in 12 well culture plates overnight. BMM were preloaded with LysoTracker Red (1∶10,000; Molecular probes, Carlsbad, California, USA) for 1 h and infected with *F. tularensis* subsp. *novicida* as described above. 30 min after infection, cells were washed and treated with gentamicin for 30 min. Next, cells were washed again, incubated for additional 2 h and processed for confocal laser microscopy. In brief, after fixation, cells were permeabilized with 0.1% Triton X-100/PBS (Zheng et al 2003) for 15 min followed by incubation with blocking buffer (5% goat serum in PBS) for 2 h. The cells were incubated with mouse anti-*F. tularensis* subsp. *novicida* monoclonal antibody at a 1∶100 dilution for 4 h followed by the addition of Alexa Fluor 488 goat anti-mouse IgG for 2 h. After washing with PBS, the nuclei were labeled with DNA stain 4, 6-diamidino-2-phenylindole (DAPI) for 5 min at room temperature. The coverslips were mounted using ProLong Gold Antifade reagent. At least 50 cells per sample (collected from random fields) were analyzed using a Zeiss Laser Scanning Microscope LSM 510 to score the co-localization of *Francisella* and LysoTracker. Three independent, blinded evaluations were performed for each sample.

### Microscopy Analysis of *Francisella phagocytosis*

Phagocytosis of *Francisella* was evaluated by microscopy as we have previously described with a few modifications [19]. In brief, 60 minutes post-infection, cells were washed with PBS and fixed in 4% paraformaldehyde for 20 minutes. The samples were divided into two. One set of cells was permeabilized with 100% methanol for 10 minutes. Immunostaining was performed with mouse anti-*F. tularensis* subsp. *novicida* LPS antibody (diluted 1/100; Immune Precise Antibodies) and the bacteria were visualized by anti-mouse Alexa Fluor 488 secondary antibody. Bacteria attached to macrophages were counted on the non-permeabilized samples and total number of bacteria associated (both attached and phagocytosed) with the cells were counted on methanol permeabilized samples using an X100 oil immersion objective of a BX40 Olympus fluorescence microscope. The number of bacteria phagocytosed was obtained by subtracting the number of bacteria attached to the cell from the total number of bacteria associated with the cell. At least 100 cells per sample were examined and three separate sets of infections were analyzed. All evaluations were performed in a blinded fashion. Phagocytic and attachment indices are defined as the number of bacteria phagocytosed or attached to 100 macrophages, respectively. Statistical analysis was performed by paired Student's *t-test*. *p*<0.05 was considered significant.

### Analysis of Fas (CD95) Expression by Flow Cytometry

RAW 264.7 cells were plated in 6 well tissue culture plates, pretreated with mithramycin A (50 nmole) or vehicle control DMSO for 30 minutes and infected with *F. tularensis* subsp. *novicida*. After infection, cells were scraped and incubated in blocking buffer (5% goat serum in PBS) for 10 minutes on ice to block Fcγ receptors. The cells were incubated with Fas (CD95) antibody (Abcam, Cambridge, MA, USA) or an isotype control antibody in blocking buffer for one hour on ice, followed by staining with FITC-conjugated secondary antibody (Molecular Probes, Carlsbad, CA, USA). The cells were then washed with PBS and fixed with 1% paraformaldehyde. Fluorescence-labeled cells were analyzed on a FACScan using the CellQuest software package (Becton Dickinson).

### Sp-1/Sp-3 Luciferase Assay

RAW 264.7 cells were transfected with the Sp-1/Sp-3-luciferase reporter construct (Sp-1/Sp-3 luc) using the Amaxa Nucleofector apparatus (Amaxa biosystems, Germany). Transfected cells were either left uninfected or were infected with *F. tularensis* subsp. *novicida*. Cells were lysed in 100 µl of Luciferase Cell Culture Lysis Reagent (Promega). Luciferase activity was then measured using Luciferase Assay Reagent (Promega) as previously described22.

### Statistical Analysis

Data were analyzed by either a Student's t-test or ANOVA using the R.2.6.1 software (http://www.r-project.org).

## Results

### *F. tularensis* Subsp. *novicida* Infection Induces Fas Expression and Activates the Fas-Mediated Death Pathway

**Figure 1:**
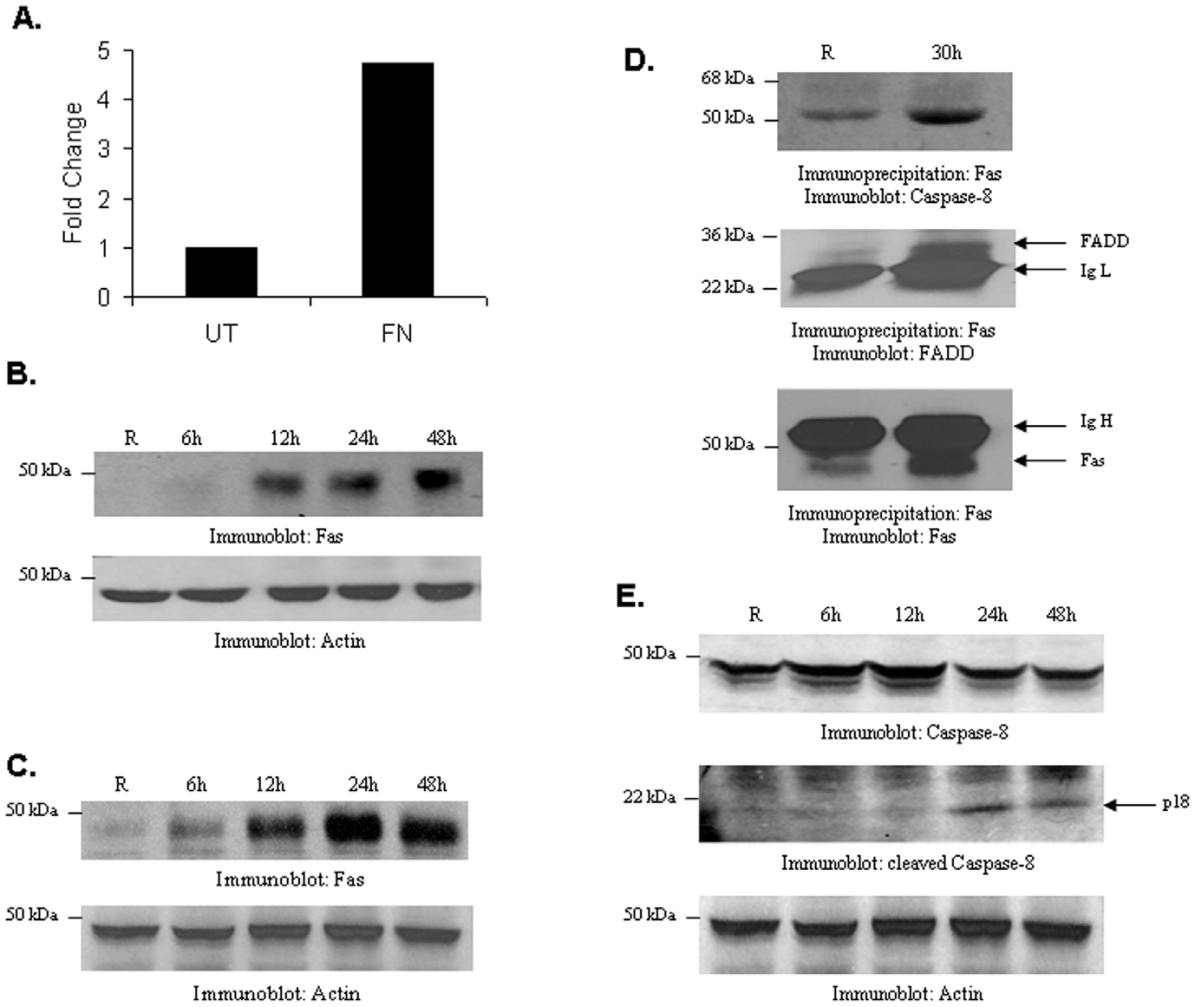
*F. tularensis* subsp. *novicida* induces Fas expression and activation of the Fas-mediated death pathway. (**A**) BMM were infected with *F. tularensis* subsp. *novicida* (MOI 100) for 24 h. RNA was isolated and analyzed by microarray. Fold change in Fas expression between untreated and *F. tularensis* subsp. *novicida* infected cells is shown. (**B**) BMM were infected and analyzed by Western blotting with Fas antibody. The lower panel is a reprobe with actin antibody (**C**) RAW 264.7 cells were infected and analyzed for Fas expression by Western blotting. (**D**) RAW 264.7 cells were infected, immunoprecipitations were performed with Fas antibody and analyzed for the presence of the DISC components by Western blotting with caspase-8 and FADD antibodies. The membranes were reprobed with Fas antibody. IgH, IgG heavy chain; IgL, IgG light chain (**E**) RAW 264.7 were infected and analyzed for caspase-8 activation by Western blotting. The upper panel shows the full length caspase-8 and the middle panel shows cleaved Caspase-8. **B–E** were performed three times, with similar results.

Murine bone marrow derived macrophages (BMM) were infected with *F. tularensis* subsp. *novicida* for 24 h and RNA was extracted and analyzed for global gene expression by GeneChip® Mouse Genome 430 2.0 Arrays. From the array data we found that the death receptor Fas (CD-95) expression was enhanced over 4.5 fold in infected cells (Fig. 1A). Fas protein expression was verified by Western blotting protein-matched lysates from BMM infected with *F. tularensis* subsp. *novicida* for various time points. Results indicated that the expression of Fas protein is dramatically induced in infected cells (Fig. 1B). A similar induction of Fas expression was observed in RAW 264.7 cells infected with *F. tularensis* subsp. *novicida* (Fig. 1C).

We next investigated whether the Fas-mediated death pathway was activated in infected cells. Fas-FasL engagement results in the formation of the death-inducing signaling complex (DISC) which involves the recruitment of FADD and procaspase-8 to the receptor [30], [31]. To test for the formation of DISC upon infection with *F. tularensis* subsp. *novicida*, Fas immunoprecipitates from infected and uninfected RAW 264.7 cells were analyzed for the presence of FADD and caspase-8 by Western blotting. The results shown in Figure 1D demonstrate that Fas associates with both FADD and caspase-8 in infected cells. Finally, we also examined whether caspase-8 was cleaved in infected cells. As shown in Figure 1E, caspase-8 cleavage is apparent in infected cells around 24 h post infection. Together, these results indicate that *F. tularensis* subsp. *novicida* infection induces the expression of Fas and activates the Fas-mediated death pathway. Next, the expression of Fas was analyzed in macrophages infected with different MOI (1∶1, 10∶1 and 100∶1) of bacteria, and the results revealed that Fas expression levels increased with increasing MOI (data not shown). In addition, we analyzed whether bacterial viability had an effect on Fas expression. For this, RAW 264.7 cells were infected with live or heat-killed bacteria and analyzed for Fas expression by Western blotting. Results revealed that bacterial viability is required in order to increase the Fas expression in macrophages (data not shown).

### Phagosomal Escape of *F. tularensis* Subsp. *novicida* Regulates Expression of Fas and Activation of Caspase-3

**Figure 2:**
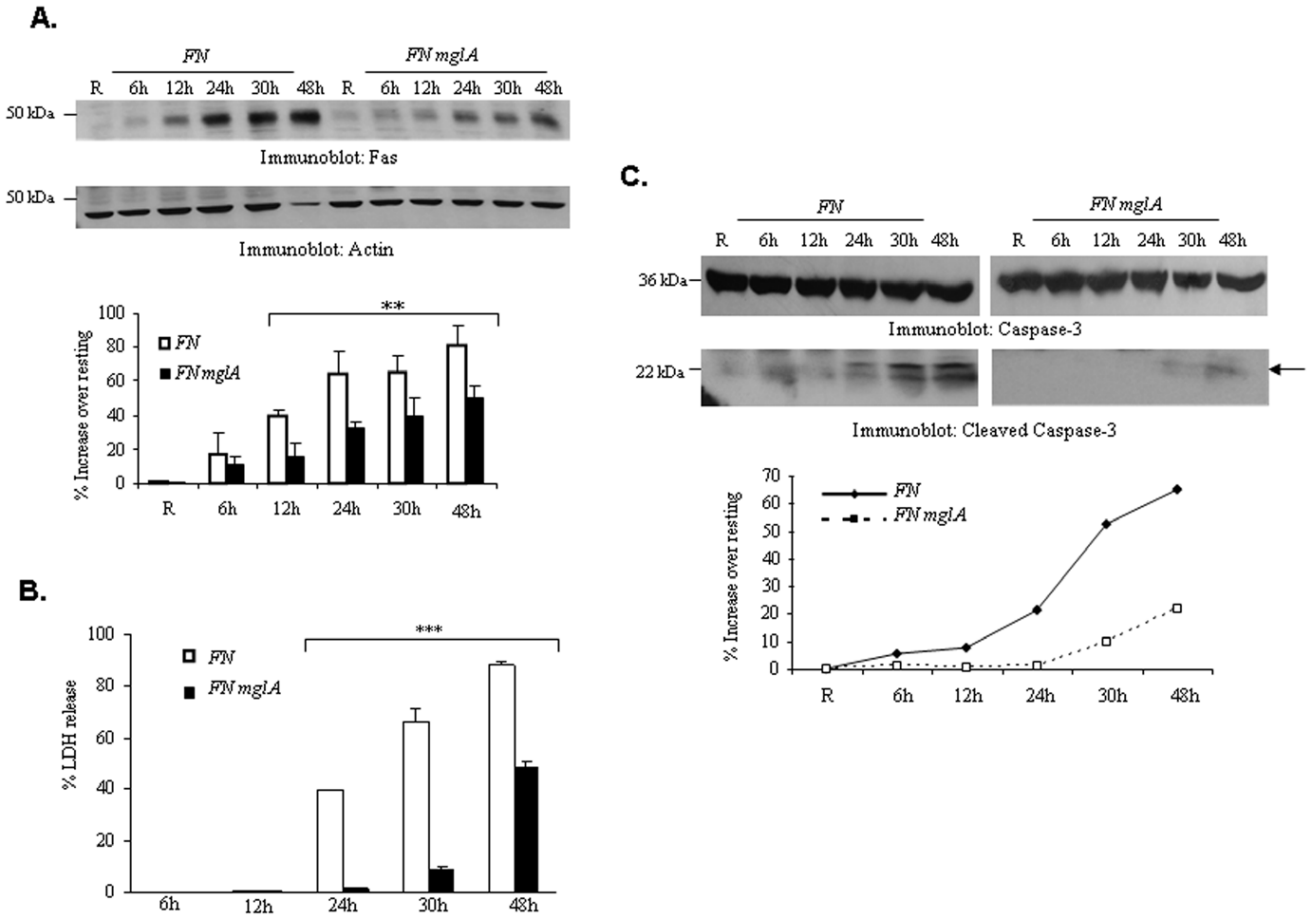
Phagosomal escape is essential for *F. tularensis* subsp. *novicida*-induced up-regulation of Fas expression and Caspase-3 activation. RAW 264.7 was infected with wild-type or a *mglA* mutant of *F. tularensis* subsp. *novicida* (*FN mglA*) for 6 h, 12 h, 24 h, 30 h and 48 h. (**A**) Cell lysates were analyzed by Western blotting with Fas antibody. The membranes were reprobed with actin antibody. The graph shows Fas band intensity values obtained from three independent experiments. * indicates p value less that 0.05. (**B**) In a parallel assay, cell death was evaluated by LDH release in RAW 264.7 cells for indicated time points (*** p<0.00039 for the comparison of wild-type and the *mglA* mutant of *F. tularensis* subsp. *novicida*). (C) Cell lysates were analyzed for caspase-3 cleavage by Western blotting. The upper panel shows intact caspase-3 and the middle panel shows cleaved caspase-3. The band intensity of cleaved caspase-3 was measured. The graphs show the mean and SD of values from three independent experiments.

*F. tularensis* subsp. *novicida* escapes from the phagosome to establish infection in macrophages. The transcriptional regulator *mglA* plays a crucial role in phagosomal escape. To verify whether the phagosomal escape of bacteria is required for the induction of Fas expression, RAW 264.7 cells were infected with wild type *F. tularensis* subsp. *novicida* or an *mglA* mutant of *F. tularensis* subsp. *novicida* (*FN mglA*). This mutant has significantly lower ability to escape from the phagosome and has been widely used to examine the influence of phagosomal escape on various host cell responses [9], [10], [16], [22]. Fas protein expression was significantly reduced in cells infected with *FN mglA* (Fig. 2A). In parallel experiments, cell supernatants were assayed for the presence of LDH as a measure of host cell death and protein-matched lysates were analyzed for caspase-3 cleavage. Both cell death, as measured by LDH release, and caspase-3 cleavage were lower in *FN mglA*-infected cells (Fig. 2B and C). To ensure that infection was equivalent in cells infected either with wild type *F. tularensis* subsp. *novicida* or *FN mglA*, intracellular bacterial load was measured by CFU assays. Results indicated that intracellular bacterial load was equivalent at 30 min post infection (data not shown). As expected the CFUs were significantly lower in FN *mglA*-infected cells at 8 h post infection, likely due to a defect in phagosomal escape and replication. These data suggest that the induction of Fas expression and apoptosis requires that the bacteria escape the phagosome. These results are consistent with a previous report in which an *iglC* mutant of *F. tularensis* LVS, which is defective in phagosomal escape, failed to induce apoptosis in J774.1 macrophages [11].

### The PI3K/Akt Pathway Regulates Fas Expression in Macrophages

**Figure 3:**
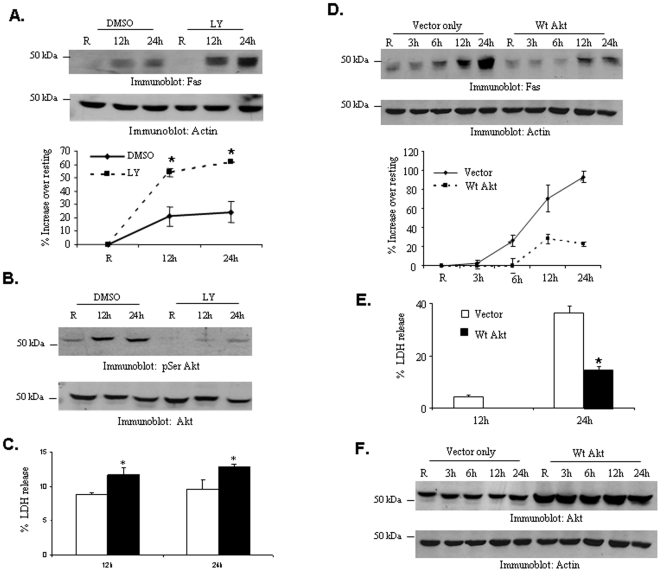
The PI3K/Akt pathway regulates Fas expression and cell death during *F. tularensis* subsp. *novicida* infection. (**A**) RAW 264.7 cells were pretreated with PI3K inhibitor LY 294002 for 30 minutes and then infected. Lysates were analyzed by Western blotting with Fas antibody. The same membranes were reprobed with actin antibody. The graph shows mean and SD of band intensity values obtained from three independent experiments. Data were analyzed by a student's t-test. * indicates p value <0.05. (**B**) Cell lysates were analyzed with phospho-Akt antibody to verify inhibition of the PI3K pathway. (**C**) Cell death was analyzed by measuring the amount of LDH release. (**D**) RAW 264.7 cells were transfected with empty vector or plasmids expressing wild-type Akt. The transfectants were infected and Fas protein expression was determined by Western blotting. The graph shows mean and SD of Fas band intensities from three independent experiments. * indicates p value <0.05. (**E**) Cell death was assessed by LDH assay. The graph shows mean and SD of LDH release from three independent infections. (**F**) Over-expression was confirmed by Western blot analysis with Akt antibody.

We have recently reported that the PI3K-Akt pathway enhances the production of host-protective cytokines during *Francisella* infection [22], although it did not influence uptake of these bacteria by murine macrophages [32].We therefore examined whether this pathway played a role in the induction of Fas in infected cells. First, the PI3K pathway was inhibited in RAW 264.7 cells using the pharmacologic inhibitor LY 294002. The cells were then infected with *F. tularensis* subsp. *novicida* and Fas induction was analyzed by Western blotting. The results indicated that inhibition of PI3K leads to a significantly enhanced expression of Fas upon infection (Fig. 3A). Notably, Fas expression was not induced in the absence of infection in the inhibitor-treated cells. To verify that the LY 294002 treatment had indeed inhibited the PI3K pathway, parallel lysates were analyzed for phosphorylation of Akt, the downstream effector of PI3K (Fig. 3B). Consistent with enhanced Fas expression, cell death (as measured by LDH release) was also significantly enhanced in infected cells treated with the PI3K inhibitor (Fig. 3C).

In an alternate approach, we over-expressed wild type Akt in RAW 264.7 cells and subsequently analyzed Fas expression and cell death in response to *F. tularensis* subsp. *novicida* infection. The results indicated that overexpression of Akt leads to significantly reduced induction of Fas and cell death in infected cells (Fig. 3D and E). Akt over-expression was verified by Western blotting with Akt antibody (Fig. 3F).

**Figure 4:**
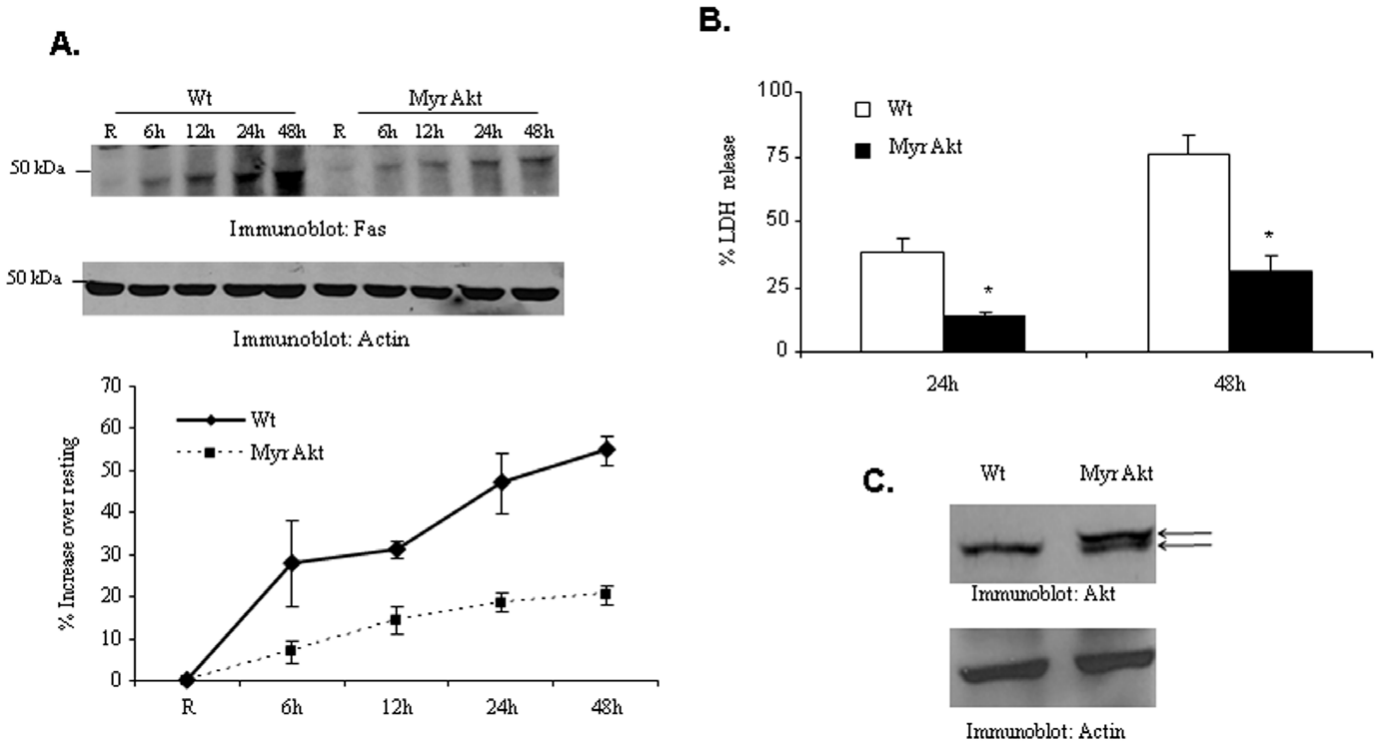
Constitutive activation of Akt in macrophages modulates Fas expression in response to *F. tularensis* subsp. *novicida* infection. (A) BMM isolated from wild type and Myr Akt mice were infected with *F. tularensis* subsp. *novicida* for the indicated time points. Protein-matched cell lysates were probed with Fas antibody and subsequently reprobed with actin antibody. The graph shows the mean and SD of Fas band intensities from three independent experiments (*** p<0.00058 for the comparison of wild-type and Myr Akt BMM). (B) Cell death of wild type and Myr Akt BMM was examined by measuring the amount of LDH release during infection (* p<0.0012 for 12 h and p<0.0015 for 24 h). The graph shows mean and SD of LDH release from three independent infections. (C) To confirm the expression of the Myr Akt transgene, cell lysates were analyzed by Western blotting with Akt antibody.

Finally, to analyze the role of Akt in Fas expression and cell death in response to *F. tularensis* subsp. *novicida* infection in primary macrophages, BMM were isolated from transgenic animals with macrophage-specific expression of constitutively-active Akt (Myr Akt) and their wild type littermates. Cells were infected with *F. tularensis* subsp. *novicida* and Fas expression was analyzed by Western blotting and cell death was assessed by measuring LDH release. Consistent with the findings above, Fas induction and cell death were significantly lower in cells expressing Myr Akt (Fig. 4A and B). To verify the expression of the Myr Akt transgene, cell lysates were analyzed by Western blotting with Akt antibody (Fig. 4C).

### SHIP Promotes *F.tularensis* Subsp. *novicida*-Induced Fas Expression in Macrophages

**Figure 5:**
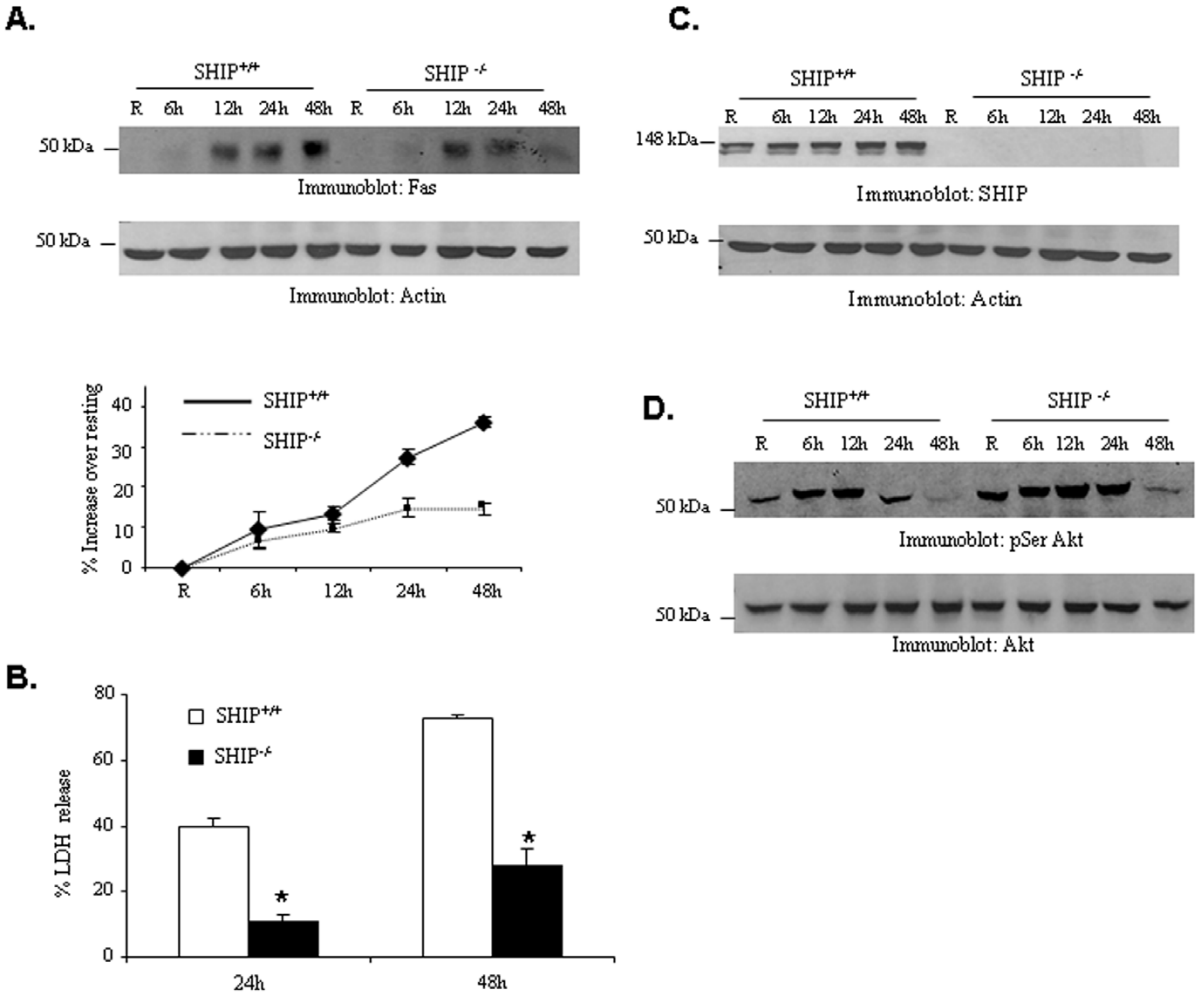
Deletion of SHIP decreases *F. tularensis* subsp.*novicida*-induced Fas expression and cell death. (**A**) BMM from SHIP^+/+^ and SHIP^−/−^ mice were isolated and infected with *F. tularensis* subsp. *novicida* for 6 h, 12 h, 24 h and 48 h. Protein-matched lysates were analyzed by Western blotting with Fas antibody (upper panel). The same membranes were reprobed with actin antibody (middle panel). The graph shows mean and standard deviation of band intensity values obtained from three independent experiments (lower panels). Data were analyzed by Student's t-test and ANOVA (** p<0.00751 for the comparison of wild type and SHIP knock out BMM). (**B**) Cell death of BMM was assessed by LDH assay. The graph shows mean and SD of LDH release from three independent infections. (**C**) The deletion of SHIP was confirmed by Western blotting with SHIP antibody. (**D**) Cell lysates were analyzed with phospho-specific antibodies for Akt.

We have recently demonstrated that the inositol phosphatase SHIP negatively regulates the PI3K/Akt pathway during *Francisella* infection [17]. This would suggest that the presence of SHIP would enhance Fas expression and subsequent cell death during infection. To test this notion, BMM from SHIP^+/+^ and SHIP^−/−^ littermates were infected with *F. tularensis* subsp. *novicida* and the induction of Fas expression was analyzed by Western blotting. The results indicated that Fas expression is significantly lower in SHIP^−/−^ BMM compared to SHIP^+/+^ BMM when infected with *F. tularensis* subsp. *novicida* (Fig. 5A). Likewise, LDH release was also significantly lower in SHIP^−/−^ BMM (Fig. 5B). The deletion of SHIP was verified by Western blotting with SHIP antibody (Fig. 5C). As previously reported [17], SHIP^−/−^ BMM displayed enhanced activation of Akt upon infection, as assessed by Western blotting with phospho-Serine Akt antibody (Fig. 5D).

### Akt Influences Phagosomal Escape of *F. tularensis* Subsp. *novicida*

**Figure 6:**
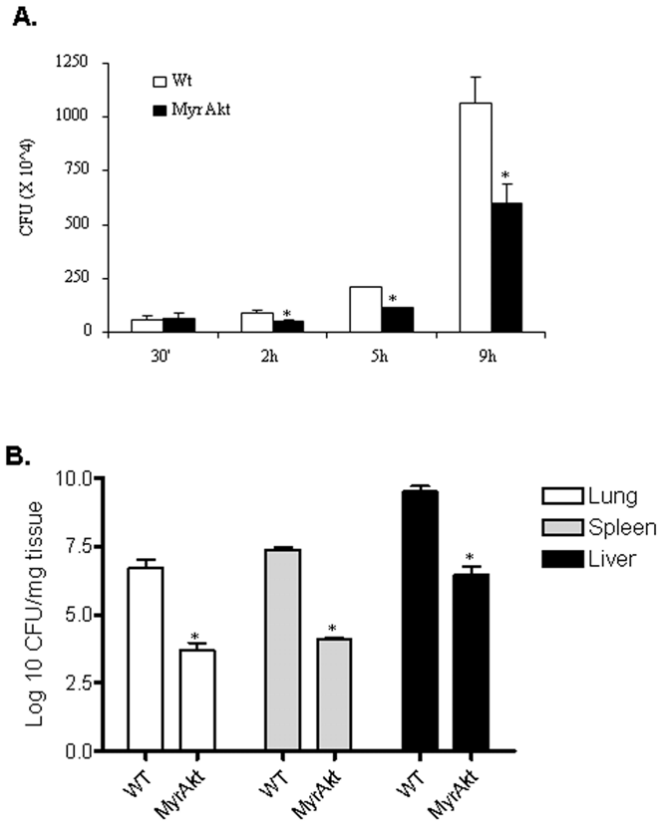
Akt influences bacterial load *in vitro* and *in vivo*. (**A**) Wild type and Myr Akt BMM were infected with *F. tularensis* subsp. *novicida*. After the indicated hours of infection, intracellular bacterial load was assessed by CFU assays. The graph shows the mean and SD of counts obtained from three independent experiments. Data were analyzed by a student's t-test. * indicates p value <0.05. (**B**) Wild type and Myr Akt mice (3 per group) were infected with *F. tularensis* subsp. *novicida* (200 CFU) by the intra-peritoneal route. After 48 h of infection the animals were sacrificed, and organs (spleen, lung and liver) were harvested and homogenized. The homogenates were analyzed for bacterial load by CFU assays. The graphs show the mean and SD of counts obtained. Data were analyzed by a student's t-test. * indicates p value <0.05.

The PI3K-Akt pathway plays an important role in cell survival. Therefore, the enhanced induction of Fas expression in infected cells pretreated with the PI3K inhibitor may be predicted. However, the findings that the expression of over-active Akt results in reduced Fas expression in infected cells has broader implications. Specifically, it indicates that the induction of host cell death is reduced despite the fact that the cells are infected with 100 MOI *F. tularensis* subsp. *novicida*, and suggests that Akt may regulate intracellular bacterial burden. Thus, we next examined the effect of Akt on intra-macrophage growth of *F. tularensis* subsp. *novicida*. For this, BMM from wild type and Myr Akt mice were infected with *F. tularensis* subsp. *novicida* for various time points. The intra-macrophage bacterial burden was analyzed by a colony forming unit (CFU) assay. We observed that at an early time point (30 minutes post infection) there was no difference in the CFUs within the macrophages derived from wild type and Myr Akt mice, whereas after 2 h of infection there was a ∼50% reduction of CFUs in the macrophages expressing constitutively-active Akt. We observed a similar trend at later time points (5 h and 9 h). These results suggest that Akt influences the clearance of bacteria and/or phagosomal escape, but not uptake of the bacteria (Fig. 6A).

To examine the influence of Akt on bacterial burden *in vivo*, age-matched wild-type and Myr Akt mice were injected with 200 cfu *F. tularensis* subsp. *novicida*, and the organs were harvested after 48 h and analyzed for bacterial load by CFU assays. All organs tested from Myr Akt mice showed a significantly lower bacterial burden compared to the organs from wild-type animals. The graphs in Figure 6B show the mean and standard deviation of values obtained from three pairs of mice for spleen, lung and liver.

### Akt Promotes the Fusion of *Francisella-*Containing Phagosomes (FCP) with Lysosomes

**Figure 7:**
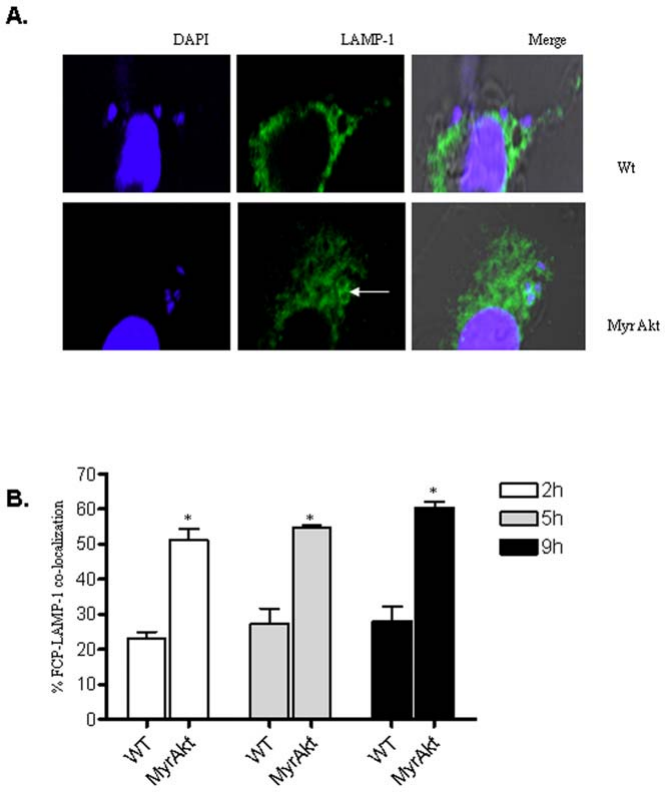
Akt promotes co-localization of the *Francisella*-containing phagosome (FCP) and the late endosomal-lysosomal protein LAMP-1. (**A**) BMM from wild type and Myr Akt mice were infected with *F. tularensis* subsp. *novicida* at an MOI of 100 for 30 min, and the extracellular bacteria were killed and removed by gentamicin treatment followed by washing. The infected cells were incubated for 2 h and then fixed as described in Materials and Methods. The preparation was incubated with LAMP-1 antibody followed by Oregon Green 488 anti-rat IgG antibody. The bacteria and macrophage nuclei were stained with DAPI for 5 minutes at room temperature. The FCP-lysosome colocalization was examined using a LSM 510 confocal microscope (magnification ×26000). (**B**) The number of FCP-lysosome co-localization events was scored at varying time points post infection. LAMP-1 co-localization was scored positive only when a complete ring of intensified LAMP1 staining was detected around the bacterium. The graph shows the mean and SD of three independent experiments. 100 cells were scored in each experiment. * indicates a p value <0.05.

To determine whether Akt influences FCP-lysosome fusion, synchronized *F. tularensis* subsp. *novicida* infections were performed in wild type and Myr Akt BMM, extracellular bacteria were removed by gentamicin treatment 30 min post infection and the infected cells were further incubated for 2 h, 5 h and 9 h. Maturation of the FCP was analyzed based on the acquisition of the late endosomal marker protein LAMP-1 (lysosomal-associated membrane protein-1) around the bacteria. The number of FCP- LAMP-1 co-localization events was scored by confocal microscopy (Fig. 7A). In wild-type macrophages, only 23% of the FCP co-localized with LAMP-1, whereas in Myr Akt macrophages greater than 51% of the FCP were co-localized with LAMP-1 at 2 h time point, indicating that activation of Akt promotes FCP-LAMP-1 co-localization. Kinetic experiments showed a persistence of significantly higher FCP-LAMP-1 co-localization events in the Myr Akt-expressing BMM at 5 h and 9 h post infection (Fig. 7B).

**Figure 8:**
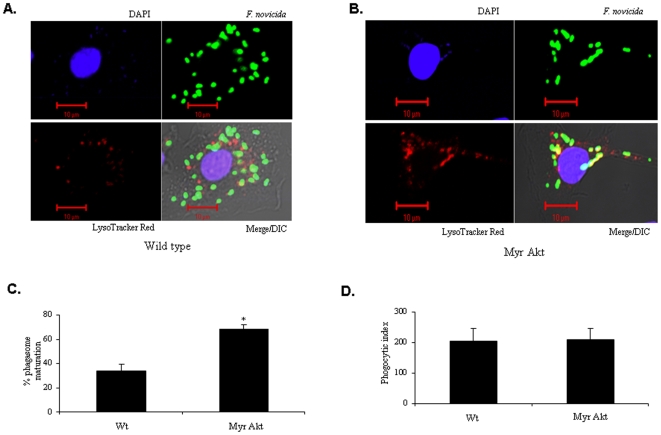
Akt promotes the colocalization of FCP with lysosomes. BMM from wild type (**A**) and Myr Akt (**B**) mice were pre-loaded with LysoTracker red for 30 min and subsequently infected with *F. tularensis* subsp. *novicida* at an MOI of 100 for 30 min. The extracellular bacteria were killed and removed by gentamicin treatment followed by washing. The infected cells were incubated for 2 h and then fixed. The preparation was incubated with mouse anti-*F tularensis* subsp. *novicida* monoclonal antibody followed by the addition of Alexa Fluor 488 goat anti-mouse IgG. The macrophage nuclei were stained with DAPI for 5 min at room temperature. The FCP (green)-lysosome (red) colocalization was examined using a LSM 510 confocal microscope (magnification ×26000). (**C**) The number of FCP-lysosome co-localization events was scored. Positive only when FCP fused completely with lysosomes (orange). The graph shows mean and SD of three independent experiments. 100 cells were scored in each experiment. * indicates p value <0.05. (**D**) In a parallel experiment the phagocytic index (uptake of bacteria) was analyzed by immunofluorescence microscopy. BMM were infected with *F. tularensis* subsp. *novicida* at a MOI of 100. After 30 min of infection, cells were fixed and immunostained with *F. tularensis* subsp. *novicida* LPS antibody followed by Alexa Fluor 488-conjugated secondary antibody. At least 100 cells per sample were examined and three separate sets of infections were analyzed. The graph shows the mean and SD of three independent experiments. * indicates a p value <0.05.

In a second approach, FCP-lysosome fusion was analyzed by using LysoTracker red which selectively labels acidic lysosomes. BMM from wild-type and Myr Akt mice were loaded with LysoTracker Red and infections were performed following the synchronized and pulse chase methods. Cells were fixed and bacteria were labeled with *F. tularensis* subsp. *novicida* LPS antibody followed by a FITC conjugated secondary antibody. The co-localization of bacteria with lysosomes was scored in 100 cells. The experiment was repeated thrice and analyzed in a blinded fashion. Results showed that the number of FCP-lysosome fusion events in wild-type BMM was only 33% compared to that in Myr Akt-expressing macrophages (68%) (Fig. 8A–C). However, the uptake of bacteria by wild type and Myr Akt-expressing macrophages was equal (Fig. 8D). Together these data indicate that Akt promotes the fusion of FCP with lysosomes.

### SHIP Suppresses FCP-Lysosomal Fusion and Promotes Intra-Macrophage Growth

**Figure 9:**
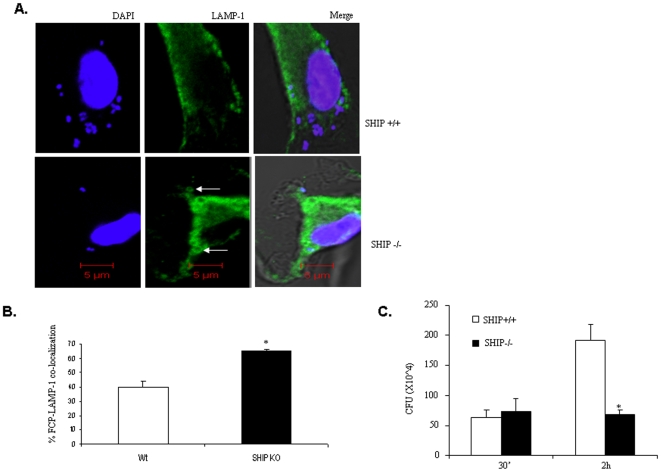
SHIP regulates FCP-LAMP-1 co-localization. (**A**) SHIP^+/+^ and SHIP^−/−^ BMM were infected with *F. tularensis* subsp. *novicida*. Infected cells were prepared for confocal microscopy as described in Figure 7. LAMP-1 co-localization was scored positive only when a complete ring of intensified LAMP1 staining was detected around the bacterium. (**B**) The graph shows the mean and SD of values obtained from three independent experiments. In each experiment 100 cells were scored. (**C**) Intracellular bacterial load of *F. tularensis* subsp. *novicida* in SHIP^+/+^ and SHIP^−/−^ BMM was analyzed by CFU assays. The graph shows the mean and SD of three independent experiments performed in triplicate. * indicates a p value <0.05.

**Figure 10:**
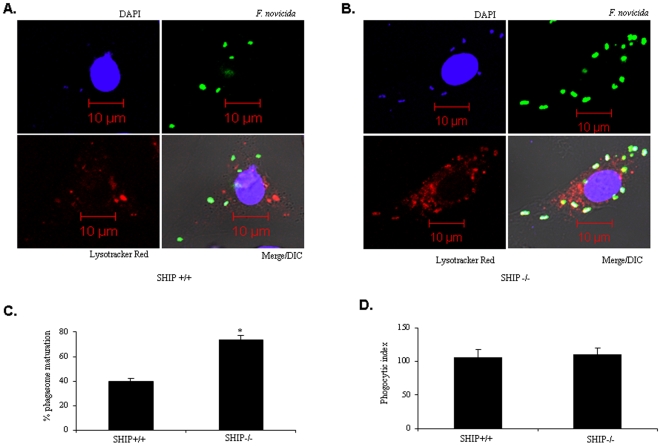
SHIP regulates FCP-lysosome co-localization. (**A**) SHIP^+/+^ and (**B**) SHIP^−/−^ BMM were infected with *F. tularensis* subsp. *novicida*. Infected cells were prepared for confocal microscopy as described in Figure 8. Co-localization was scored positive only when FCP were completely fused with lysosomes (**C**) The graph shows mean and SD of values obtained from three independent experiments. In each experiment 100 cells were scored. (**D**) The bacterial uptake in SHIP^+/+^ and SHIP^−/−^ BMM was analyzed by immunofluorcence microscopy. The graph shows mean and SD of three independent experiments performed in triplicate. * indicates p value <0.05.

We next examined the influence of SHIP on bacterial clearance. Here, BMM from SHIP^+/+^ and SHIP^−/−^ littermates were infected with *F. tularensis* subsp. *novicida*. Cells were treated with gentamicin 30 min post infection to kill the extracellular bacteria and incubated further for 2 h. The number of FCP-lysosome fusion events was scored by confocal microscopy. As shown in Fig. 9A and B, there was a significantly greater number of FCP-lysosome fusion events in SHIP^−/−^ BMM compared to SHIP^+/+^ BMM. Similar results were obtained in experiments using the LysoTracker dye to mark lysosomes (Fig. 10).

In parallel experiments, we also determined the intracellular growth of bacteria in BMM by CFU assays. Results indicated that there was no significant difference in the number of bacteria in SHIP^+/+^ and SHIP^−/−^ BMM at 30 minutes post infection, indicating that SHIP did not influence the uptake of bacteria (Fig. 9C). However, the intra-macrophage CFUs were significantly higher in SHIP^+/+^ BMM at 2 h hours post infection, suggesting that a greater number of bacteria have been able to escape phago-lysosomal fusion.

Taken together these results suggest that although phagocytosis of *Francisella* is not influenced by Akt and SHIP, these two enzymes modulate FCP-lysosome fusion and therefore intra-macrophage bacterial burden.

### Sp-1/Sp-3 Transcriptional Factors Regulate the Expression of Fas

**Figure 11:**
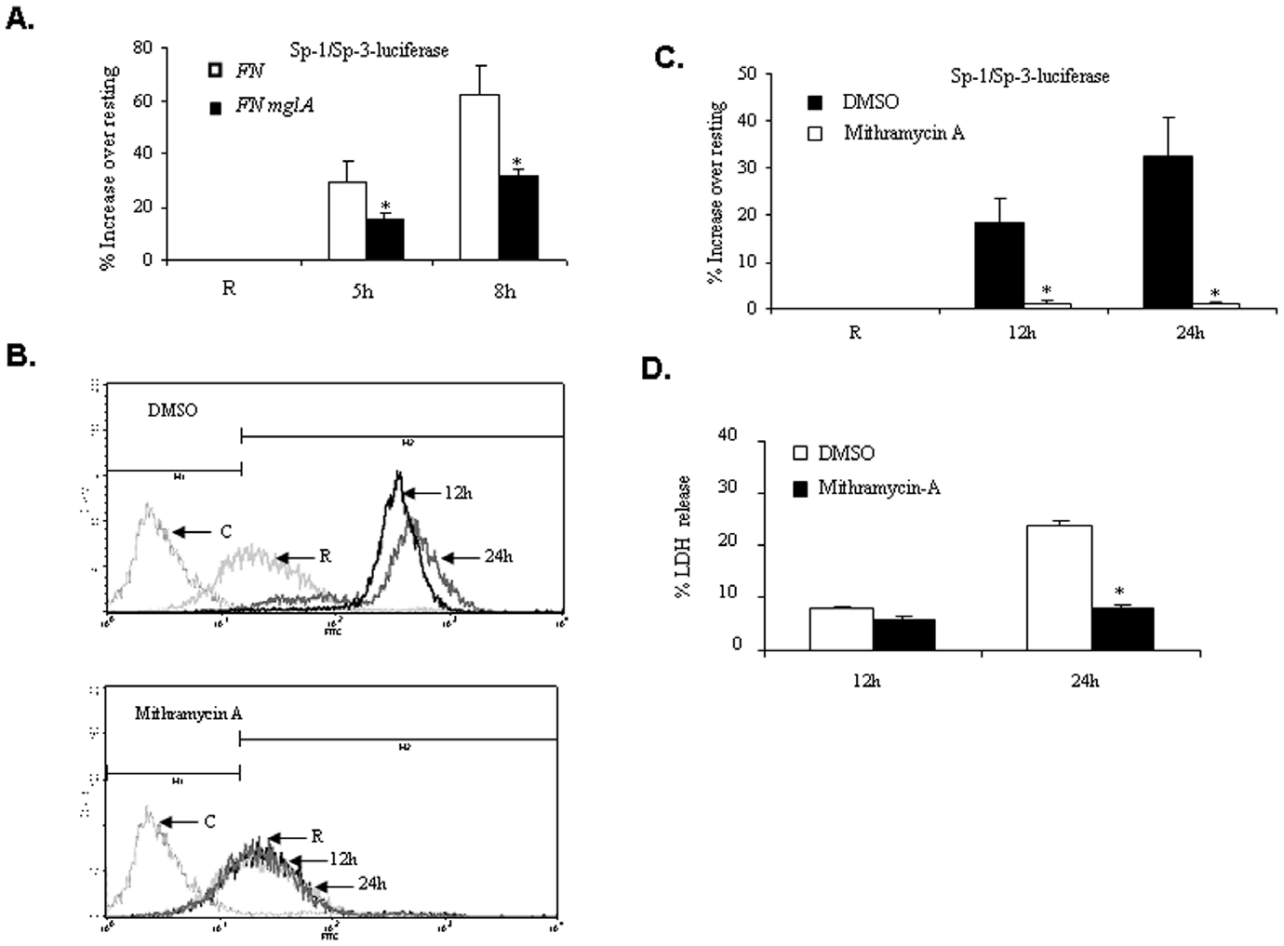
Phagosomal escape triggers the activation of Sp-1/Sp-3 leading to Fas induction and cell death. (**A**) The role of phagosomal escape on Sp-1/Sp-3 transcriptional activation was assessed by luciferase assays. RAW 264.7 cells were first transfected with Sp1/Sp3-luciferase reporter plasmid, infected with wild-type or *FN mglA* at a MOI of 100. Cell lysates were analyzed for luciferase acitivty. The graph represents the mean and standard deviation of three independent experiments performed in triplicate. * indicates a p value <0.05. (**B**) Raw 264.7 cells were pre-treated with mithramycin A (Sp-1/Sp-3 inhibitor) or vehicle control (DMSO) for 30 min, and subsequently infected with *F. tularensis* subsp. *novicida* for the indicated time points. Fas expression was assessed by flow cytometry. (**C**) In a parallel experiment, the inhibition of Sp-1/Sp-3 by mithramycin A was analyzed using the reporter assay. The graph represents the mean and standard deviation of three independent experiments performed in triplicate. * indicates p value <0.05. (**D**) Cell death was assessed by LDH assay. The graph shows mean and SD of LDH release from three independent infections.

Our data thus far show that Akt and SHIP regulate phagosomal escape and that phagosomal escape results in the induction of Fas expression and host cell death. We next examined the mechanism by which phagosomal escape of *F. tularensis* subsp. *novicida* induces Fas expression. Earlier studies have demonstrated a critical role for Sp-1/Sp-3 transcription factors in the induction of Fas [33], [34]. To determine whether Sp-1/Sp-3 were involved in the expression of Fas in infected macrophages, RAW 264.7 cells were transfected with a Sp-1/Sp-3-luciferase reporter construct and the transfectants were infected with either *F. tularensis* subsp. *novicida* or *FN mglA*. Results showed that Sp-1/Sp-3 activity was significantly lower in cells infected with *FN mglA*, indicating that phagosomal escape is necessary (Fig. 11A). In a parallel experiment, Sp-1/Sp-3 activity was inhibited with mithramycin A [35] and Fas expression was analyzed by flow cytometry. Inhibition of Sp-1/Sp-3 significantly dampened the expression of Fas induced by *F. tularensis* subsp. *novicida* infection (Fig. 11B). The inhibition of Sp-1/Sp-3 activity by mithramycin A was verified by luciferase reporter assays (Fig. 11C). Consistently, mithramycin A treatment also dramatically reduced cell death in infected cells (Fig. 11D).

**Figure 12:**
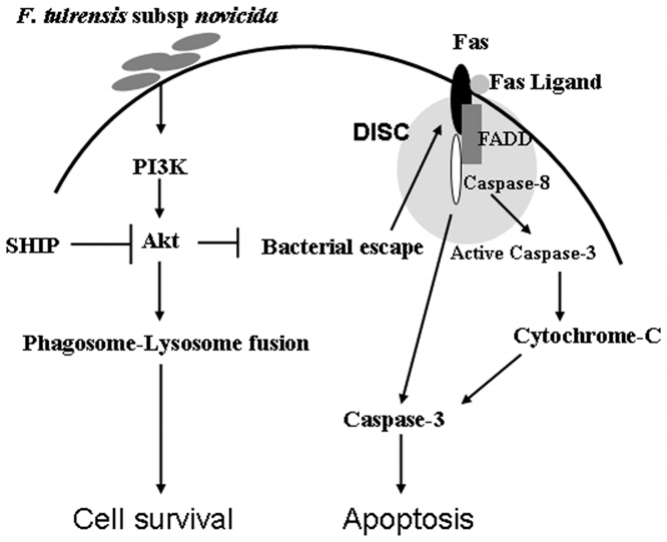
PI3K/Akt and SHIP regulate phagosome-lysosome fusion and host-cell death. After entry into macrophages *F. tularensis* subsp. *novicida* escapes the phagosome, which leads to Fas up-regulation and recruitment of the DISC complex. This activates caspase-8 and caspase-3 leading to host-cell death. PI3K/Akt promotes phagosome-lysosome fusion and therefore limits intracellular bacterial growth and host-cell death. SHIP opposes the effects of the PI3K/Akt pathway.

Taken together these results indicate that although phagocytosis of *Francisella* is not influenced by Akt and SHIP, these two enzymes modulate FCP-lysosome fusion and therefore the intra-macrophage bacterial burden. This subsequently results in the regulation of activation of Sp-1/Sp-3 transcription factors, leading to the induction of Fas expression and host cell death (modeled in Figure 12).

## Discussion

Our current study demonstrates that the Fas-mediated death pathway is activated in *Francisella*-infected cells. Although previous studies had established that caspase-1 and caspase-3-dependent apoptosis is induced in infected cells, the upstream events triggering apoptosis were not known. Our studies provide evidence that the induction of apoptosis is at least in part due to the upregulation of Fas expression. A significant induction of Fas mRNA was also observed in human monocytes infected with *F. tularensis* subsp. *novicida*, although no significant changes were seen in the expression of the Fas ligand (data not shown). Fas/Fas ligand expression and subsequent Fas-mediated apoptosis of host cells is induced during infections by other pathogens such as *Staphylococcus aureus* [36], *Helicobacter pylori* [37], [38] and HIV-1 [39]–[41].

Currently it is not conclusively known if host-cell death during *Francisella* infection is to the benefit of the host or to the pathogen. However, the importance of caspase-3 in host cell death is supported by a recent report showing that *F. tularensis* subsp. *tularensis* infection results in extensive host-cell death through a caspase-3 dependent mechanism and not caspase-1 [42]. Therefore it seems that caspase-3 activation is a major factor in the pathogenesis of tularemia, so understanding the regulation of caspase-3 activity is critical.

The induction of Fas in our infection model is negatively regulated by the PI3K/Akt pathway and positively regulated by SHIP. Interestingly, the manipulation of these pathways in the absence of infection did not induce Fas expression. This suggests that the influence of these pathways on the induction of the Fas-mediated death pathway is likely indirect. Consistent with this, our data show that Akt and SHIP modulate FCP-lysosomal fusion and hence bacterial load in the host cell. This in turn modulates the activation of the transcription factors Sp-1/Sp-3, leading to the induction of Fas expression and host cell death. In line with this conclusion, phagosomal escape was required for increased activation of Sp-1/Sp-3 and Fas induction. Given the timing of events for bacterial escape, replication, and activation of Sp-1/Sp-3, it seems logical that Fas expression only begins to appear at 6 hours post-infection, though continues to increase over the course of infection in wild-type macrophages that do not effectively control intracellular bacteria.

The ability of *Francisella* to escape the phagosome and replicate in the host cell cytosol is a critical feature of its intracellular life style. Bacterial proteins that are responsible for phagosomal escape are extensively studied. However, prior to this work there was little information of host cell signaling machinery that might influence this critical event. Previous studies demonstrated a role for class III PI3K and its product PI3P, but not class I PI3K in phago-lysosomal fusion during *Salmonella* infection [43], [44]. Our current findings that there is enhanced FCP-lysosomal fusion and reduced bacterial load in cells expressing either over-active Akt or lacking SHIP indicate a role for class I PI3K and its phosphoinositide products in the maturation of the FCP. There are several possible explanations for the influence of Akt and SHIP on FCP-lysosomal fusion. A role for class I PI3K and specifically Akt in phagosome maturation has been demonstrated by Rupper et in *Dictyostelium* [45]. It was proposed that PI3K activity may be involved in regulating phagosomal pH and that Akt may stimulate the activation of Rab5. The latter notion comes from the studies of Barbieri et al. where the blockade of endosomal fusion by a dominant-negative Akt was reversed by the expression of constitutively-active Rab5 [46]. Another reason that Akt activation may promote phagosome lysosome fusion is through the enhanced activation of NFκB. We have previously shown that Akt promotes NFκB activation [22] and in the case of mycobacteria it has been shown that NFκB controls phagosome fusion-mediated killing of bacteria [47]. While the NFκB-dependent effector proteins were not precisely identified, it was clear that NFκB activation was critical for intra-macrophage killing of bacteria. Alternately, it is possible that Akt promotes the oxidative burst and killing of *Francisella* within the phagosome, thus causing the bacteria to fuse with the lysosome. Akt has been shown to phosphorylate phox proteins to promote the assembly of the NADPH oxidase complex [48], [49]. We are currently investigating these possibilities.

We have previously reported that Akt activation is dampened by phagosomal escape of *Francisella*. We also demonstrated that the down-regulation of Akt activation results in reduced production of host-protective cytokines such as IL-12 [22]. Our current findings indicate that suppression of Akt activation can also lead to reduced FCP-lysosomal fusion and enhanced bacterial survival in the host cell. Thus it appears that the bacteria may have developed strategies to suppress the PI3K/Akt pathway to promote their own survival. Previous reports have shown that intracellular pathogens such as *Salmonella* produce the effector protein SigD/SopB which can hydrolyze phosphoinositides such as PI 3,4,5P_3_ and thereby regulate the PI3K pathway [50]. A similar strategy may be utilized by *Francisella* to interfere with host cell phosphoinositide signaling to gain an advantage over the host. While this currently remains a mere possibility, there is ample evidence to show suppression of this pathway in a recent report by our lab exploring global changes in host response genes between *F. tularensis* subsp. *tularensis* and *F. tularensis* subsp. *novicida* infections. We found that the expression of the PI3K/Akt pathway members was preferentially down-regulated with the highly virulent *F. tularensis* subsp. *tularensis* [51]. So impairment of the PI3K/Akt pathway may contribute to the high virulence of *F. tularensis* subsp. *tularensis*. Uncovering the mechanisms through which the highly virulent subspecies of *Francisella* suppress the PI3K/Akt pathway should help understand the pathogenic potential of this organism.

In conclusion, we report that phagosomal escape/intracellular bacterial burden and induction of the Fas-mediated death pathway are regulated by the opposing actions of PI3K/Akt and the inositol phosphatase SHIP. To our knowledge, this is the first description of modulation of the intracellular niche of *Francisella* by host cell signaling pathways.
